# Diagnostic suspicion bias and machine learning: Breaking the awareness deadlock for sepsis detection

**DOI:** 10.1371/journal.pdig.0000365

**Published:** 2023-11-01

**Authors:** Varesh Prasad, Baturay Aydemir, Iain E. Kehoe, Chaya Kotturesh, Abigail O’Connell, Brett Biebelberg, Yang Wang, James C. Lynch, Jeremy A. Pepino, Michael R. Filbin, Thomas Heldt, Andrew T. Reisner

**Affiliations:** 1 Harvard-MIT Program in Health Sciences and Technology, Massachusetts Institute of Technology, Cambridge, Massachusetts, United States of America; 2 Institute for Medical Engineering and Science, Massachusetts Institute of Technology, Cambridge, Massachusetts, United States of America; 3 Department of Emergency Medicine, Massachusetts General Hospital, Boston, Massachusetts, United States of America; 4 Department of Electrical Engineering and Computer Science, Massachusetts Institute of Technology, Cambridge, Massachusetts, United States of America; Massachusetts Institute of Technology, UNITED STATES

## Abstract

Many early warning algorithms are downstream of clinical evaluation and diagnostic testing, which means that they may not be useful when clinicians fail to suspect illness and fail to order appropriate tests. Depending on how such algorithms handle missing data, they could even indicate “low risk” simply because the testing data were never ordered. We considered predictive methodologies to identify sepsis at triage, before diagnostic tests are ordered, in a busy Emergency Department (ED). One algorithm used “bland clinical data” (data available at triage for nearly every patient). The second algorithm added three yes/no questions to be answered after the triage interview. Retrospectively, we studied adult patients from a single ED between 2014–16, separated into training (70%) and testing (30%) cohorts, and a final validation cohort of patients from four EDs between 2016–2018. Sepsis was defined per the Rhee criteria. Investigational predictors were demographics and triage vital signs (downloaded from the hospital EMR); past medical history; and the auxiliary queries (answered by chart reviewers who were blinded to all data except the triage note and initial HPI). We developed L2-regularized logistic regression models using a greedy forward feature selection. There were 1164, 499, and 784 patients in the training, testing, and validation cohorts, respectively. The bland clinical data model yielded ROC AUC’s 0.78 (0.76–0.81) and 0.77 (0.73–0.81), for training and testing, respectively, and ranged from 0.74–0.79 in four hospital validation. The second model which included auxiliary queries yielded 0.84 (0.82–0.87) and 0.83 (0.79–0.86), and ranged from 0.78–0.83 in four hospital validation. The first algorithm did not require clinician input but yielded middling performance. The second showed a trend towards superior performance, though required additional user effort. These methods are alternatives to predictive algorithms downstream of clinical evaluation and diagnostic testing. For hospital early warning algorithms, consideration should be given to bias and usability of various methods.

## Author summary

Predictive algorithms for hospitals often rely on the results of diagnostic tests as predictors for whether patients have serious and unexpected conditions. Strong predictive performance of such algorithms might be misleading for the following reason: doctors may not order the appropriate diagnostic tests unless they already have some level of concern about the patient, so the data will be available if doctors are already suspecting the correct diagnosis but not available in cases when doctors overlook the correct diagnosis. In this manuscript, we consider early sepsis identification and explore two alternative strategies for avoiding any reliance on diagnostic testing: the use of “bland” data that should be available on every single patient, and the use of a few objective “yes/no” questions that might be answered on patients with abnormal vital signs, to provide additional information for the predictive algorithms.

## Introduction

Substantial efforts have focused on machine learning (ML) algorithms for automated identification of illness, [1] although adoption has generally been slower than hoped [2,3]. Sepsis detection is an exemplary topic because early identification could enable earlier treatment and better outcomes [4].

Numerous reports described promising sepsis detection algorithms, [5] although prospective performance has repeatedly fallen short [6,7]. Further editorializing about one such report [7], the senior author provided non-peer reviewed commentary that the sepsis detection algorithm had used antibiotic orders as a key predictive input, allowing for strong retrospective performance but inferior prospective performance due to incorporation bias [8]. Incorporation bias occurs when the investigational predictors are also determinative factors in defining the outcome [9]. Population drift is a second factor that has been cited to explain reduced prospective performance [10].

A third potential factor has not received as much attention: diagnostic suspicion bias. In general, predictive algorithms for hospitalized patients categorize the results of diagnostic testing. Yet certain diagnostic tests may not be performed until after clinicians already have evaluated a patient, developed diagnostic suspicion, and ordered appropriate tests [11]. In multiple reports, the availability of such diagnostic tests has been associated with illness–*independent* of the actual diagnostic results–because clinicians perform testing precisely because they are concerned about a patient. The frequency of vital-sign checks, [12] the frequency of blood tests, [13] and the sending of bloodwork in the middle of the night have all been correlated with illness [14]. Conversely, clinicians may not perform timely testing without *a priori* diagnostic concern, and it is in this situation when decision support from a predictive model could be most useful.

This poses a major challenge for early warning algorithms that rely on diagnostic testing data. Depending on how an algorithm handles missing data, an algorithm could indicate that the patient is low risk *simply whenever there are no diagnostic data to suggest otherwise*. The clinician might then observe the algorithm’s erroneous “low-risk” prediction, take false reassurance, and so further delay appropriate testing. In other words, an awareness deadlock between clinician and computer could arise: a feedback loop reinforcing the other’s failure to suspect the correct diagnosis. In this scenario, the early warning algorithm would in fact be worse than nothing, because it would actively reinforce a clinician’s error in diagnostic judgement. The risks of an awareness deadlock may be widespread, considering that a review article of 107 predictive algorithms found that *none of them* had accounted for so-called “informative observations” (i.e., when the presence or absence of a diagnostic observation was not random) [1].

Our goal was to explore a predictive algorithm for sepsis identification for an overcrowded Emergency Department (ED), where there could be long waits for many patients before any evaluation or testing. In this scenario, the ideal sepsis prediction algorithm would not be downstream of diagnostic testing; instead, the ideal algorithm would provide early identification of patients who should receive the early evaluation and/or treatment without waiting for diagnostic testing to be completed. Prior reports have found that septic patients often present to EDs with vague symptoms and without obvious vital-sign abnormality, and that these were the patients most at risk of antibiotic treatment delay [15,16].

In reviewing the published literature, we did not find broadly accepted best practices to minimize diagnostic suspicion bias for early warning algorithms. Our team decided to explore two strategies. The first was to rely only on “bland clinical data” which were data elements that should be available on nearly every patient at triage, regardless of clinicians’ suspicions of illness. The second strategy involved “auxiliary queries” in which clinicians would be prompted to answer brief, objective questions that augmented bland hospital data. Neither strategy required diagnostic testing results. In this report, we explore these strategies by developing exemplary algorithms, and we discuss their operational implications.

## Results

### Patient population

From Interval-1, we analyzed 1,663 patients in total, 1,164 (70%) of which constituted the training set while 499 (30%) were reserved as a hold-out test set. From Interval-2, we studied an additional 784 patients (sixteen patients excluded for missing basic vital signs, i.e., temperature or RR). The subject characteristics for subjects from the primary hospital Massachusetts General Hospital (MGH) are provided in Table 1 and Table 2, broken down by Interval and by non-sepsis versus sepsis cases. Subject characteristics for subjects from the other hospitals are available in S1 File. Median Cohen’s kappa for various parameters determined by chart review was 0.76 (interquartile range 0.68 to 0.85).

**Table 1:** Patient characteristics. Values are presented as median (interquartile range) or proportion of cohort.

	Training Cohort		Testing Cohort		MGH Validation Cohort	
	Non-sepsis Cases (N = 590)	Sepsis Cases (N = 574)	Non-sepsis Cases (N = 245)	Sepsis Cases (N = 254)	Non-sepsis Cases (N = 131)	Sepsis Cases (N = 66)
**Demographics**						
Age, years	58 (39, 71)	67 (55, 77)	59 (39, 71)	64 (53, 76)	60 (46, 74)	67 (59, 77)
Male, %	49	60	47	56	51	56
**Race**						
American Indian or Alaska Native, %	<1	0	0	0	<1	0
Asian, %	4	5	4	3	3	6
Black or African American, %	8	5	5	6	8	6
Middle Eastern or Northern African, %	<1	2	0	0	0	0
Native Hawaiian or Pacific Islander, %	0	0	<1	0	0	2
Other, %	8	6	10	5	5	8
White or Caucasian, %	74	78	76	83	77	78
Unavailable, %	4	4	3	2	4	0
**Ethnicity**						
Hispanic, %	5	4	4	2	8	10
Non-Hispanic, %	87	88	92	96	88	84
Unavailable, %	9	8	3	2	4	6
**Past medical history**						
Coronary artery disease, %	16	24	18	21	21	41
Congestive heart failure, %	16	21	16	21	21	17
Chronic kidney disease, %	11	24	15	26	21	24
Chronic obstructive pulmonary disease, %	12	18	15	16	13	30
Cerebrovascular accident, %	7	11	9	8	9	24
Liver disease, %	4	9	6	9	6	5

MGH, Massachusetts General Hospital.

**Table 2:** Characteristics of Emergency Department presentation and outcomes. Values are presented as median (interquartile range) or proportion of cohort.

	Training Cohort		Testing Cohort		MGH Validation Cohort	
	Non-sepsis Cases (N = 590)	Sepsis Cases (N = 574)	Non-sepsis Cases (N = 245)	Sepsis Cases (N = 254)	Non-sepsis Cases (N = 131)	Sepsis Cases (N = 66)
**Vital signs**						
Triage SBP, mmHg	117 (103, 137)	111 (92, 133)	116 (100, 136)	112 (89, 138)	127 (108, 151)	118 (99, 133)
Proportion of patients w/ ED SBP <90 mmHg, %	28	75	30	69	29	59
Median time to hypotension from triage, min	N/A	55 (0, 212)	N/A	41 (0, 185)	N/A	93 (16, 301)
Triage Heart rate, bpm	92 (75, 111)	107 (89, 121)	87 (75, 105)	110 (90, 124)	92 (77, 105)	99 (84, 112)
Triage Glasgow Coma Scale score	15 (15, 15)	15 (13, 15)	15 (14, 15)	15 (14, 15)	15 (15, 15)	15 (15, 15)
Triage respiratory rate, min^-1^	18 (18, 20)	20 (18, 22)	18 (18, 20)	20 (18, 24)	20 (18, 20)	20 (18, 22)
Triage SpO_2_, %	98 (96, 99)	96 (94, 98)	98 (95, 100)	96 (93, 98)	97 (95, 99)	97 (94, 98)
Triage temperature,°F	97.7 (97.0, 98.4)	98.0 (97.1, 99.5)	97.6 (97.0, 98.3)	98.0 (97.0, 99.4)	97.5 (96.9, 98.4)	98.0 (97.3, 98.9)
**ED diagnostics**						
First serum lactate, mmol/L	1.8 (1.2, 4.3)	3.4 (2.1, 5.4)	2.0 (1.2, 4.6)	4.0 (2.4, 5.3)	1.5 (1.1, 2.5)	2.8 (1.6, 4.3)
No Lactate, %	51	1	50	2	44	2
Sent < 1 hour, %	30	71	31	67	5	39
Sent 1 to 3 hours, %	13	20	13	21	36	44
Sent ≥ 3 hours, %	6	8	7	11	15	15
White blood cell count, 1000/μL	9.5 (6.9, 13.3)	13.8 (7.6, 19.3)	9.1 (6.6, 13.7)	13.3 (7.4, 18.6)	9.4 (7.7, 12.6)	13.7 (8.2, 18.9)
No WBC, %	11	<1	12	<1	10	0
Sent < 1 hour, %	49	78	51	72	37	44
Sent 1 to 3 hours, %	30	19	27	20	40	42
Sent ≥ 3 hours, %	10	2	9	7	13	14
**Outcome**						
Infection source, %						
Pulmonary	N/A	24	N/A	27	N/A	30
Urinary		20		16		26
Intraabdominal		19		24		24
Skin / soft tissue		5		8		9
Other		6		5		0
Unknown		23		19		6
Hospital mortality, %	11	25	11	24	2	23

MGH, Massachusetts General Hospital.

### Model composition

After parameter selection, the Bland Model consisted of nine physiologic and demographic variables (Triage oxygen saturation [SpO_2_]; high temperature; low temperature; SBP; Glasgow Coma Scale score; shock index; respiratory rate; gender; age).

After parameter selection, the Essential Model included the same parameters as in the Bland model (except it no longer included low temperature); and the Essential Model also included the responses to each of the three auxiliary queries, and a single true-false indicator of whether the patient had at least one major comorbidity.

After parameter selection, the Full Model consisted of 24 variables: age; ten different past medical history conditions; five symptoms; six vital signs; and two elements from the history of present illness.

Additional details and descriptions about these investigational parameters are available in S1 File. Additional technical details about the model are also available in the first author’s doctoral thesis [17].

### Model performances

ROC AUCs are provided in Table 3. The following observations are offered:

- The ROC AUC for qSOFA trended below all investigational models. This was apparent for every cohort and sub-cohort. In some cases, the 95% confidence interval (CI) of the qSOFA was below the 95% CI of the investigational models;
- The ROC AUCs for the Bland Model generally trended lower than the Essential Model and the Full Model;
- Despite fewer input parameters, the Essential Model yielded similar ROC AUCs to the Full Model;
- Overall, for each model, ROC AUCs were similar through all cohorts and sub-cohorts. In other words, within each individual column of Table 3, ROC AUCs were generally consistent.

**Table 3:** Area under the receiver operating characteristic curve (ROC AUC) for identification of sepsis upon ED triage by four investigational prediction models.

Cohort	qSOFA Model	Bland Model	Essential Model	Full Model
**Training set (MGH)**	0.66 (0.63, 0.69)	0.78 (0.76, 0.81)	0.84 (0.82, 0.87)	0.86 (0.84, 0.88)
Training set random sub-cohort	0.61 (0.52, 0.70)	0.76 (0.68, 0.84)	0.81 (0.74, 0.87)	0.80 (0.73, 0.86)
**Hold-out test set**	0.63 (0.59, 0.68)	0.77 (0.73, 0.81)	0.83 (0.79, 0.86)	0.82 (0.78, 0.86)
Hold-out test set random sub-cohort	0.60 (0.48, 0.73)	0.73 (0.61, 0.85)	0.80 (0.68, 0.92)	0.80 (0.71, 0.90)
**Validation cohorts**				
**MGH**	0.58 (0.50, 0.66)	0.74 (0.68, 0.81)	0.79 (0.73, 0.85)	
MGH random sub-cohort	0.75 (0.59, 0.90)	0.91 (0.84, 0.99)	0.81 (0.68, 0.94)	
**BWH**	0.65 (0.58, 0.73)	0.79 (0.73, 0.85)	0.83 (0.77, 0.88)	
BWH random sub-cohort	0.55 (0.32, 0.78)	0.94 (0.88, 1.0)	0.92 (0.85, 0.99)	
**NWH**	0.64 (0.56, 0.73)	0.74 (0.67, 0.81)	0.78 (0.72, 0.85)	
NWH random sub-cohort	0.28 (0, 0.86)	0.68 (0.22, 1.0)	0.84 (0.55, 1.0)	
**NSMC**	0.59 (0.50, 0.68)	0.77 (0.70, 0.84)	0.80 (0.74, 0.87)	
NSMC random sub-cohort	0.79 (0.54, 1.0)	0.96 (0.9, 1.0)	0.96 (0.89, 1.0)	

Results in parentheses are the 95% confidence intervals for the ROC AUCs. qSOFA has been recommended by the Surviving Sepsis Campaign for early recognition of sepsis. The Bland Model only used data typically available on all patients at triage, i.e., age and vital signs. The Essential Model consisted of the same parameters as in the Bland Model, in addition to the responses to each of the three auxiliary queries, and a single true-false indicator of whether the patient had at least one major comorbidity. The Full Model was developed from all data elements available at triage, including granular coding of the patient’s past medical history and of the patient’s reported symptoms. BWH, Brigham and Women’s Hospital; MGH, Massachusetts General Hospital; NSMC, North Shore Medical Center; NWH, Newton Wellesley Hospital.

Additional details about model performance are available in S1 File and in the first author’s doctoral thesis [17].

Diagnostic test characteristics for the Essential Model are provided in Table 4, exploring diagnostic test performance at one lower threshold (i.e., higher sensitivity) and one higher threshold (i.e., higher specificity). The lower threshold was intended to offer sepsis screening, i.e., sensitivity > 80%. The higher threshold was intended to indicate patients who were likely (>50% PPV) to have sepsis. The findings shown in Table 4 suggest that the Essential Model may be more useful for sepsis screening at triage instead of classifying which patients actually do have sepsis. Specifically, test characteristics using the threshold ≥ 0.2 were encouraging for a screening test, whereas there were worse test characteristics, including weak F1 scores, when classifying patients using the high-specificity threshold ≥ 0.6.

**Table 4:** Diagnostic test characteristics for the Essential Model using triage vital signs.

Model threshold	Hospital	Sensitivity	Specificity	PPV	NPV	Accuracy	F1
≥ 0.2	MGH	80%	61%	51%	86%	68%	62%
	BWH	91%	66%	62%	92%	76%	74%
	NWH	75%	68%	48%	87%	70%	58%
	NSMC	69%	72%	45%	88%	71%	54%
	*Average*	*79%*	*67%*	*52%*	*88%*	*71%*	*62%*
≥ 0.6	MGH	36%	90%	65%	74%	72%	47%
	BWH	49%	84%	64%	73%	70%	55%
	NWH	25%	87%	44%	75%	70%	32%
	NSMC	31%	90%	50%	80%	75%	38%
	*Average*	*35%*	*88%*	*56%*	*76%*	*72%*	*43%*

BWH, Brigham and Women’s Hospital; ED, emergency department; HR, heart rate; ICU, intensive care unit; MGH, Massachusetts General Hospital; NSMC, North Shore Medical Center; NWH, Newton Wellesley Hospital. PPV, positive predictive value; NPV, negative predictive value.

### Audit for biases related to social determinants of health

In multivariable analysis of the relationship between the Essential Model output and sepsis, race/ethnicity and gender were non-significant predictors, i.e., p > 0.05, indicating that there was no statistically significant global bias towards positive predictions nor negative predictions by the Essential Model as a function of race/ethnicity nor gender. Examining the Essential Model’s prediction accuracy at both the high-sensitivity and high-specificity cut-offs, there was no increased prediction error associated with non-white/Hispanic nor non-male patients. Additional details of this audit for bias are provided in S1 File.

## Discussion

Diagnostic suspicion bias is a theoretical risk of early-warning machine learning algorithms that analyze in-hospital clinical data. Depending on how they handle missing data, such algorithms could predict “low risk” simply when there were insufficient diagnostic tests. Then, the clinician might delay testing because of false reassurance from the algorithm, instigating a “diagnostic deadlock.” In such cases, the predictive algorithm could exacerbate a diagnostic delay and be worse than nothing.

Diagnostic suspicion bias is probably an inherent risk of most existing sepsis early identification algorithms, because almost all use laboratory data and repeated vital sign measurements as inputs [5]. Furthermore, one review article found that 0-out-of-107 clinical prediction algorithms accounted for “informative observations” (i.e., when the presence or absence of a diagnostic observation was not random), [1] which suggests that the topic is generally under-appreciated. In one paper, Delahanty *et al*. reported an impressive ROC AUC of 0.93 to 0.97 for ED sepsis prediction. Here is how the algorithm of Delahanty *et al* handled missing data:

“*[w]e replaced unobserved data points with an extreme value (–9*,*999)*. *In our experience*, *extreme values indicating the absence of a feature produce better performance than other approaches for handling unobserved data*.” [18]

In other words, that algorithm imputed an *impossibly* reassuring lactate result of –9,999 whenever clinicians did not already suspect sepsis enough to send a lactate. This may be close to a predictive algorithm determining that if the clinician did not check a lactate, then the patient must not have sepsis. Such an assumption may lead to better performance, but it will not help recognize sepsis before the clinicians have enough concern to order tests.

In this report, we explored predictive algorithms that did not rely on clinical suspicion and diagnostic testing. We developed the Bland Model which only relied on data available for nearly all patients at triage. Unsurprisingly, using only bland data as inputs to the model yielded middling predictive performance (ROC AUC 0.77; 95% CI: 0.68–0.84 in the MGH validation dataset). A similar vital-signs-plus-demographics sepsis prediction model described by Horng *et al*. also had unimpressive performance (ROC AUC 0.67) [19]. Overall, it seems that a model based on such limited input data can offer only non-specific performance. As a practical matter, such a model would translate to some combination of frequent false alarms and/or poor sensitivity. Perhaps the best use of bland data algorithms is to suggest when clinicians should consider sending additional diagnostic testing that allows for better predictive performance (e.g., “consider sending serum lactate to screen for sepsis” or “consider recheck of vital signs within the next hour”).

If bland hospital data are too non-specific, another approach we evaluated was the use of objective yes/no auxiliary queries. These queries are analogous to conventional clinical decision rules. For example, the PERC rule for pulmonary embolism asks objective questions such as whether a patient has unilateral leg swelling. In principle, using auxiliary queries that can be answered objectively at triage could enable better algorithm performance without reliance on diagnostic testing. Indeed, we found that our “Essential Model” trended toward improved AUCs in all cohorts (*note*: in this exploratory paper, we did not formally assess statistical significance of these differences).

It is worth noting that both the Essential Model and the Bland Model performed better in the random cohorts, based on higher ROC AUCs. By contrast, when datasets were supplemented with additional potential sepsis cases, i.e., patients with hypotension and antibiotic treatment, there was a trend towards worse ROC AUCs, especially for the Bland Model which predicted sepsis solely on the basis of triage vital signs. [Note: This was likely explained because selecting for ED patients with hypotension and antibiotic treatment yielded two related sub-cohorts: *i*) truly septic patients; and *ii*) hypotensive patients who were treated for bacterial infection in the ED but did not ultimately meet the formal Rhee criteria for sepsis. It was likely more challenging for the classifiers to predict sepsis after the datasets were supplemented with a substantial number of patients who appeared septic yet did not meet sepsis criteria].

Of note, all investigational models clearly outperformed the qSOFA score, which is the detection algorithm recommended by the Surviving Sepsis Campaign [20]. Improved sepsis prediction offers the prospect of reducing antibiotic administration delays, which has been associated with reduced mortality at our institution [21] and in a range of other reports [22].

Another key issue identified for predictive algorithms is dataset shift [10]. Dataset shift occurs when the relationship between input parameters and the predicted outcome changes through time. In essence, dataset shift represents a form of overfitting to early datasets. We observe that predictive performance of our complex “Full Model” did degrade from its training set to its hold-out test set. On the other hand, the simpler Essential Model showed consistent performance in testing versus validation, despite the passage of two years, the roll-out of a new EMR, and the advent of US CMS SEP-1 quality measures [23]. This illustrates the old dictum that increased model complexity raises the risk of overfitting and reduced external validity.

**Fig 1:**
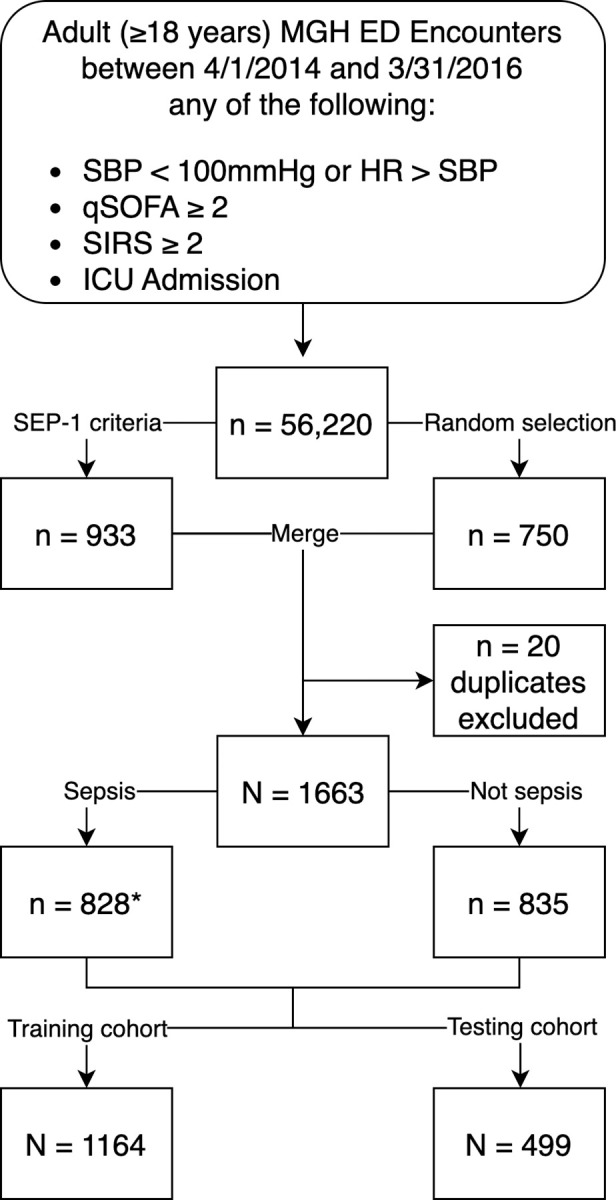
Selection of encounters for inclusion in Interval-1. Subjects from Interval-1 included randomly selected subjects plus additional patients who met the CMS SEP-1 criteria for sepsis. We excluded n = 20 of encounters who were duplicates. The presence or absence of sepsis was determined using the Rhee sepsis criteria. ED, emergency department; HR, heart rate; ICU, intensive care unit; MGH, Massachusetts General Hospital; qSOFA, quick sequential organ failure assessment; SBP, systolic blood pressure; SEP-1, Center for Medicare and Medicaid Services severe sepsis/septic shock bundle performance measure; SIRS, systemic inflammatory response syndrome. * n = 57 encounters from random selection with sepsis.

Finally, for any predictive model, it is important to consider biases associated with social determinants of health, including race/ethnicity and gender. Although the inputs to the investigational predictive models seem objective datapoints, there are well-established biases in how accurately such diagnostic data are measured [24] including racial biases involving pulse oximetry [25] and temperature [26]. To this end, we evaluated whether there were any independent associations between the Essential Model and race/ethnicity and gender as predictors of sepsis and did not find any. We also did not find that non-white/Hispanic nor non-male patients were more likely to have “errors” in prediction. On the other hand, there remains the possibility that our original inclusion criteria (see Fig 1) may have led to some form of bias in upstream subject selection, and there may be biases in the data that underlie the Rhee sepsis criteria.

There are other potential limitations to consider. Firstly, we only evaluated a logistic regression model using greedy-forward feature selection. Our intent was to explore the feasibility of sepsis prediction solely with information available upon triage (i.e., vital signs and a few interview questions), to avoid any reliance on diagnostic testing results. We explored these exemplary classifiers over two multi-year time intervals and across four different hospitals. This analysis establishes a proof-of-principle and benchmarks for classifier performance. Future investigation should consider additional classification methods, and consider how to further optimize performance, especially for the “high specificity” thresholds. Secondly, although we included patients from four hospitals—including two urban medical centers and two community hospitals—our patients were all sourced from a single geographic region. As the literature demonstrates, performance of sepsis prediction algorithms can vary in different settings. Thirdly, our randomly selected cohorts did not contain a large fraction of septic patients, and so we artificially added additional patients who were likely septic based on other EMR query criteria (as detailed in the Methods section). It is possible that there was bias in our criteria for finding those additional septic patients, i.e., additional septic patients were not truly representative of actual septic patients. It is notable that the ROC AUCs for the true random sub-cohorts (which included truly randomly selected septic patients) were at least as good, and this suggests that any bias from adding those additional septic patients was at worst a minor factor. Fourthly, our auxiliary query questions were answered by blinded chart reviewers, rather than actual clinicians treating patients. In practice, clinicians may suffer “*pop-up fatigue*” and fail to accurately respond to the auxiliary query. Perhaps auxiliary queries would be most practical if minimized with optimized trigger criteria; kept as simple as possible; and suppressed if sufficient data are already available. The Bland Model could be used to trigger the auxiliary questions pop-up when there is elevated sepsis risk.

In summary, algorithms that rely on suspicion-dependent inputs may provide false reassurance precisely when sepsis isn’t already suspected, plausibly causing delays in testing and diagnosis. This potential bias appears to be underappreciated in many prior reports. We proposed two alternative approaches to avoid this risk. While both alternatives may carry some downside (non-specific performance for algorithms using bland hospital data only; and pop-up fatigue for auxiliary queries), they may be preferable to biased algorithms with potential harm to patients. This analysis is intended as a case study to raise awareness about diagnostic suspicion bias and illustrate potential strategies to address the issue.

## Methods

### Setting and participants

Under local IRB approval, this research study was conducted with a waiver of informed consent as per US 45CFR46.116(d). We retrospectively studied adult (≥18 years) patients who were treated in EDs of our medical system. Patients were eligible if they had *any one* of the following documented *at any time during their ED stay*: systolic blood pressure [SBP] < 100 mmHg; heart rate [HR] > SBP (i.e., positive “shock index”); qSOFA score ≥ 1; [20] 2 or more SIRS criteria; [27] or admission from the ED to an intensive care unit. This selected for a pool of patients with relatively minor vital sign abnormalities and/or admission to an ICU regardless of vital signs.

Subjects were selected for Interval-1 (April 1, 2014 through March 31, 2016) for a single urban, academic ED. We randomly selected **750 patients** from Interval-1. Given the small proportion of septic patients included in this random cohort, we augmented our study population with **additional septic patients**: we included additional patients who had met CMS SEP-1 criteria for sepsis (which involved ICD-9 discharge diagnosis; this cohort had been previously analyzed by our team) [21]. The sample size for Interval-1 was determined *a priori* through Monte Carlo simulation for sufficient statistical power to estimate the area under a receiver operating characteristic curve (ROC AUC) +/- 0.05. Subjects from Interval-1 were randomly subdivided into a training cohort (70% of patients from Interval-1) and testing cohort (30% of patients from Interval-1). Subject selection for Interval-1 is described in Fig 1.

**Fig 2:**
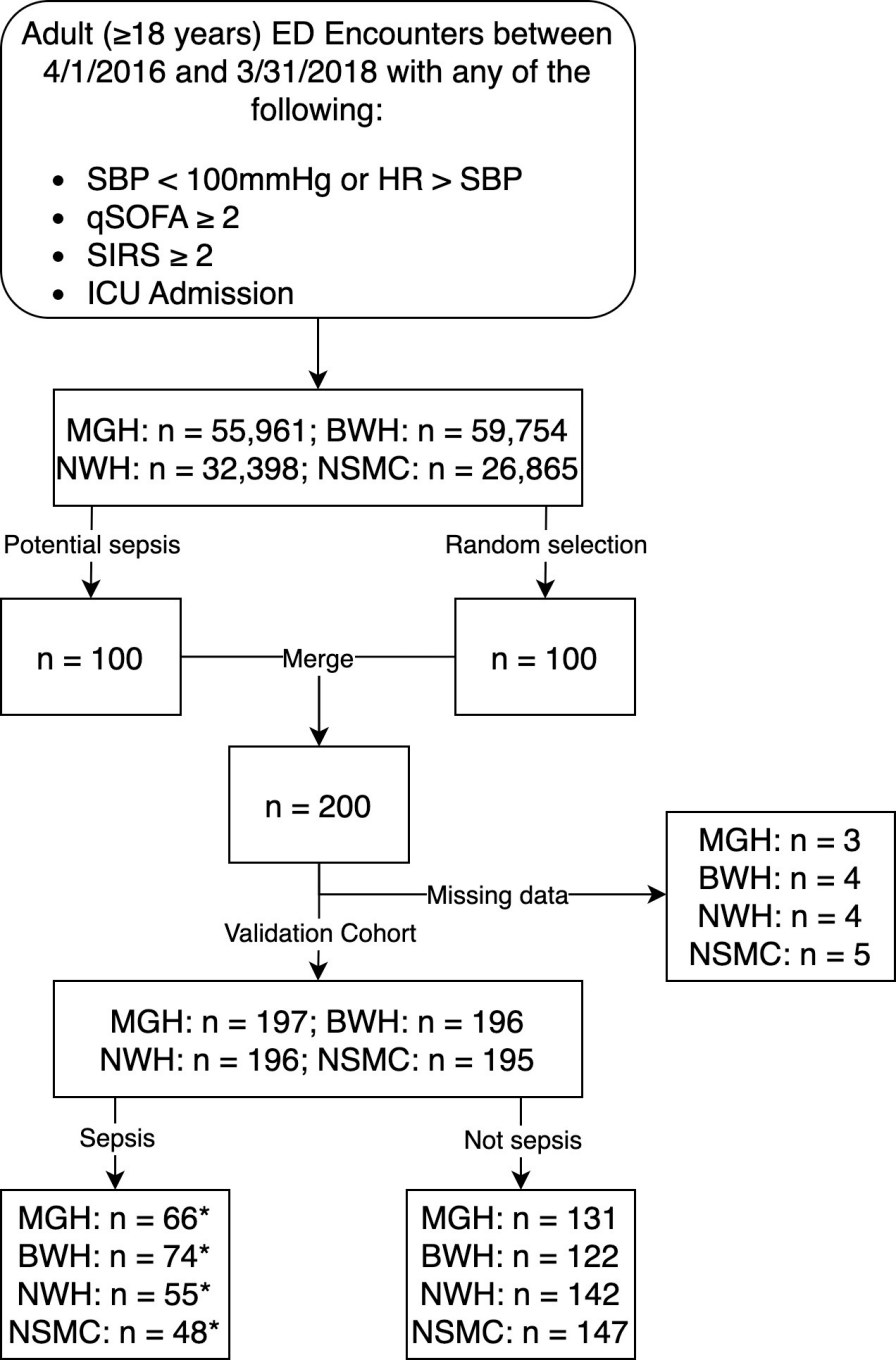
Selection of encounters for inclusion in Interval-2. Subjects from Interval-2 included randomly selected subjects plus additional patients who had hypotension documented at some time during their ED visit and also received antibiotics in the ED. Some patients were missing data necessary for calculation of the Essential Model and were excluded. The presence of absence of sepsis was determined using the Rhee sepsis criteria. BWH, Brigham and Women’s Hospital; ED, emergency department; HR, heart rate; ICU, intensive care unit; MGH, Massachusetts General Hospital; NSMC, North Shore Medical Center; NWH, Newton Wellesley Hospital; qSOFA, quick sequential organ failure assessment; SBP, systolic blood pressure; SEP-1, Center for Medicare and Medicaid Services severe sepsis/septic shock bundle performance measure; SIRS, systemic inflammatory response syndrome. *Encounters from random selection cohort with sepsis: MGH: n = 7; BWH: n = 6; NWH: n = 1; NSMC: n = 2.

After initial development and testing of the investigational sepsis prediction models, we sought additional prospective validation for multiple medical centers, including a second urban, academic ED plus two community hospital EDs, all of which have publicly reported sepsis care metrics close to both State and National Averages (see characteristics of each hospital reported in S1 File). We examined Interval-2, which started immediately after the end of Interval-1 and spanned another two years (April 1, 2016 through March 31, 2018). We randomly selected 100 subjects from each of the four hospitals’ EDs. Given the small proportion of septic patients included in this random cohort, we augmented our study population with **additional septic patients**. For Interval-2, we no longer had ready access to ICD-9 codes, because our institution switched to a new electronic data warehouse system in 2016. Therefore, to identify a cohort with high likelihood of sepsis, we selected **100 patients** with hypotension documented at some time during their ED visit who also received ED antibiotics. The sample size for Interval-2, i.e., 800 total subjects, was selected pragmatically based on our available human resources to perform chart review. Subject selection for Interval-2 is described in Fig 2.

### Variables

ICD-9/ICD-10 data were not available for Interval-2 subjects. Therefore, the study outcome, i.e., presence/absence of sepsis was determined using the Rhee sepsis criteria, [28] which only requires clinical data. We applied the Rhee sepsis criteria consistently to all subjects from both Interval-1 and Interval-2.

For investigational predictors, we analyzed “bland clinical data” that would ordinarily be available for every ED patient (triage vital signs; demographics; and past medical history elements listed in the EMR). We also evaluated the individual symptoms that were described in the triage note and the initial history of present illness (HPI). Lastly, we analyzed the responses to “auxiliary queries” which were yes/no responses to simple, objective questions:

- *Was there a report of fatigue or altered mental status*?
- *Was there a documented concern for bacterial infection prior to arrival in the ED* (e.g., referral from outpatient clinic)?
- *Was there a report of a “bacterial infection symptom complex” (BISC)*? The BISC criteria were positive if a patient has at least one localizing symptom (e.g., chest pain, flank pain, or leg pain) and at least one constitutional/inflammatory symptom (e.g., fever, or purulence). In patients with any vital-sign abnormalities, BISC criteria have been found to be specific but not sensitive for sepsis [29]. Additional details about the BISC criteria are provided in S1 File.

A detailed list of investigational predictors is provided in S1 File.

### Data sources / Measurement

Vital signs, demographics, labs, hospital medications, hospital outcome, and clinician notes were downloaded electronically from the hospital electronic data warehouse, which archives data from the electronic medical record (EMR). To confirm the validity of the downloaded data, for each parameter, at least 20 cases were randomly reviewed and compared to the subjects’ clinical data displayed in the EMR, to confirm perfect agreement, including relevant time-stamps.

Vital signs were subsequently post-processed. From the training set, we determined vital-sign cut-offs at which a monotonic association saturated, and we clipped the value of the variable at these points, determining saturation cut-offs for each parameter from the training set. For body temperature, we created variables for hyperthermia and hypothermia, separately. See S1 File for further details of the post-processing. As well, we computed the “pulse pressure” (systolic minus diastolic blood pressure) and the “shock index” (ratio of heart rate to SBP).

For the auxiliary queries, we performed blinded chart review. First, we electronically isolated the text for the triage note and the ED clinicians’ HPI and placed the records in a random order. Two independent trained reviewers, blinded to all other information (e.g., blinded to date, diagnostic results, outcome, and any subsequent clinical documentation), reviewed the triage note and HPIs. Each completed a web-based data entry form [30,31] that included whether various symptoms were present and also coded the responses to the three “auxiliary queries” after review of the clinical documentation. Completed data entry forms were compared, and disagreements resolved by a third abstractor if needed. Cohen’s kappa was computed for reviewer-coded parameters.

### Biostatistical analysis

We developed three investigational sepsis prediction models using different sets of candidate predictor features:

- The candidate predictors for the “Bland Model” were restricted to bland hospital data, i.e., the patient’s age and initial set of vital signs; this algorithm could automatically be applied to all triage patients;
- Next, we developed the “Essential Model” which used the same candidate predictors as the Bland Model; as well as a single binary indicator for the presence of any major chronic comorbidity; and the three binary auxiliary queries;
- Lastly, we developed the “Full Model” which allowed for the use of *all* investigational bland hospital data elements, including granular past medical history data elements and granular data elements extracted from chart review of the triage note and clinician HPI. The purpose of the “Full Model” was to establish an upper bound for how well sepsis could be predicted given all clinical data available at triage.

Each of these models were trained using septic and non-septic patients from the training cohort (70% of the subjects from Interval-1). Each model was developed as an L2-regularized logistic regression model, using a greedy forward feature selection approach, adding candidate features one-by-one to optimize the ROC AUC.

Each investigational model was applied to the training, testing, and validation cohort (except that the Full Model was not applied to the validation cohort because our institution changed EMR systems in 2016, including changes to how specific medical history elements were represented during 2014–2016 versus 2016–2018).

95% CI for each ROC AUC was computed using DeLong’s Method [32]. For this exploratory study, no formal biostatistical hypothesis testing was undertaken. We also applied the qSOFA score to each cohort, as a comparator. We explored the diagnostic test performance (sensitivity, specificity, and F1 score) of the “Essential Model” at two specific thresholds for the classifier: a “high-sensitivity” threshold intended as a high sensitivity screen for sepsis at the expense of some false-positives, and a “high-specificity” threshold intended to identify patients who, statistically, probably do have sepsis.

We audited results for biases that may be related to social determinants of health including race-ethnicity and gender: for all subjects in Inteval-2, we assessed whether the relationship between the Essential Model and the outcome (i.e., sepsis) was independently associated with race/ethnicity (non-white or Hispanic) or gender through multivariable analysis. Also, we assessed whether incorrect predictions by the model were associated with race/ethnicity and gender. We repeated this for both “high-sensitivity” and “high-specificity” thresholds. Additional details of this audit are provided in S1 File.

## Supporting information

S1 File Supplementary methods include details on processing continuous variables sourced from the EMR; method for adjudication of “auxiliary queries”; and method for adjudication of major comorbidities. Supplementary results include subject characteristics for additional hospitals; additional details regarding model composition; and essential model error analysis.
(PDF)
