# Supplementary material for: Diagnostic suspicion bias and machine learning: Breaking the awareness deadlock for sepsis detection
Source: PLOS Digit Health. 2023 Nov 1;2(11):e0000365. doi: 10.1371/journal.pdig.0000365 (PMC10619833; doi:10.1371/journal.pdig.0000365)
Supplement: S1 File — Supplementary results include subject characteristics for additional hospitals; additional details regarding model composition; and essential model error analysis. (PDF) [file pdig.0000365.s001.pdf]

Supplementary materials (supplementary methods and results)

Diagnostic suspicion bias and machine learning: breaking the awareness deadlock for sepsis detection

Varesh Prasad[1,2], Baturay Aydemir[3], Iain E. Kehoe[3], Chaya Kotturesh[3], Abigail O'Connell[3], Brett Biebelberg[3],

Yang Wang[3], James C. Lynch[2,4], Jeremy A. Pepino[3], Michael R. Filbin[3], Thomas Heldt[1,2,4], Andrew T. Reisner[3]

[1]Harvard-MIT Program in Health Sciences and Technology, Massachusetts Institute of Technology, Cambridge, Massachusetts, United States of America.

[2]Institute for Medical Engineering and Science, Massachusetts Institute of Technology, Cambridge, Massachusetts, United States of America.

[3]Department of Emergency Medicine, Massachusetts General Hospital, Boston, Massachusetts, United States of America.

[4]Department of Electrical Engineering and Computer Science, Massachusetts Institute of Technology, Cambridge, Massachusetts, United States of America.

Contents

1. ***Supplementary methods***

   1.1. Continuous variables sourced from the EMR or computed from such variables.

   1.2. Adjudication of “auxiliary queries”

   1.3. Adjudication of major comorbidities

2. ***Supplementary results***

   2.1. Subject characteristics for additional hospitals

   2.2. Model composition (additional details)

   2.3. Audit for biases related to social determinants of health

3. ***References***

1. Supplementary methods

1.1. Continuous variables sourced from the EMR or computed from such variables

Continuous variables sourced from the EMR or computed from such variables are listed in Table SM-1. Systolic blood pressure, diastolic blood pressure, and heart rate were normalized by their values at triage.

Table SM-1. Continuous variables (candidate predictors).

Variable
GCS
Age (years)
Temperature high
Temperature low
Pulse pressure (i.e., systolic blood pressure minus diastolic blood pressure)
Product of pulse pressure and heart rate (surrogate for cardiac output)
Mean arterial pressure divided by product of pulse pressure and heart rate (surrogate for peripheral vascular resistance)
Systolic blood pressure (mmHg)
Ratio of heart rate and systolic blood pressure (Shock index, bpm/mmHg)
SpO_2 (%)
Heart rate high (bpm)
Heart rate low (bpm)
Respiratory rate (breaths/min)

Next, we sought to accommodate major nonlinear associations between these predictors versus sepsis, using a principled, data-driven approach. For each continuously valued feature, we performed a univariate locally weighted scatter plot smoothing (LOWESS) fit to non-parametrically assess the trend of the relationship between sepsis incidence and the variable, as measured at over the whole domain of values it was observed to take on.

In general, this analysis resulted in one of three possible actions for each variable:

a) For most continuous-valued variables (for example, age), the relationship appeared to be essentially linear over the entire domain of the variable present in our data. In these cases, we incorporated these variables into the model with only a simple z-standardization.

b) The second group of variables consisted of the five variables in which there was clearly a saturation in sepsis incidence on one or both ends of the variables' domains (**Fig SM-1**, top row). For example, the incidence of sepsis clearly decreases as triage SBP increases until about an SBP of 100 mmHg. From this point and above, sepsis incidence is not associated with SBP. To build this relationship into the models, we implemented a saturation nonlinearity, clipping any variable identified to have such a relationship at the threshold value, which was identified by manual review (**Fig SM-1**, bottom row). All SBP measurements, for instance, whether at triage or later in the ED stay, were clipped such that any value above 100 mmHg was used in further analysis as exactly 100 mmHg.

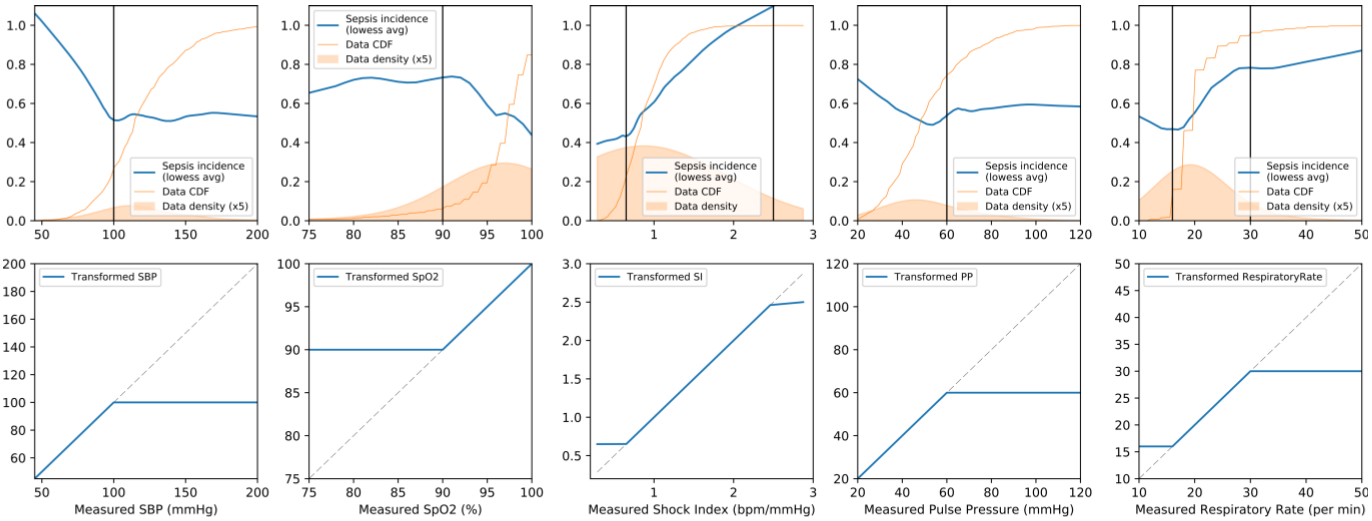

Fig SM-1: Transformations of continuous variables that were found to have monotonic associations with sepsis. Top row includes LOWESS regression with sepsis incidence, along with the cumulative distribution function of the variable at triage and a smoothed probability density function. Density functions that are very broad were multiplied by five for visualization purposed where noted. Vertical black lines indicate the thresholds below or above which variable values were clipped.

c) In the third group of variables, consisting of the five variables shown in **Fig SM-2**, we observed that the relationship with sepsis incidence was inverted at different parts of the domain (**Fig SM-2**, top row). For example, as the SIRS criteria would suggest, there is a positive association with sepsis for both abnormally elevated and abnormally decreased body temperature. To capture this biphasic relationship in the models, we transformed each variable in which we observed such a relationship into two variables, with one representing the relationship at the low end, clipped above a high threshold and the other representing the relationship at the high end, clipped below a low threshold (**Fig SM-2**, bottom row).

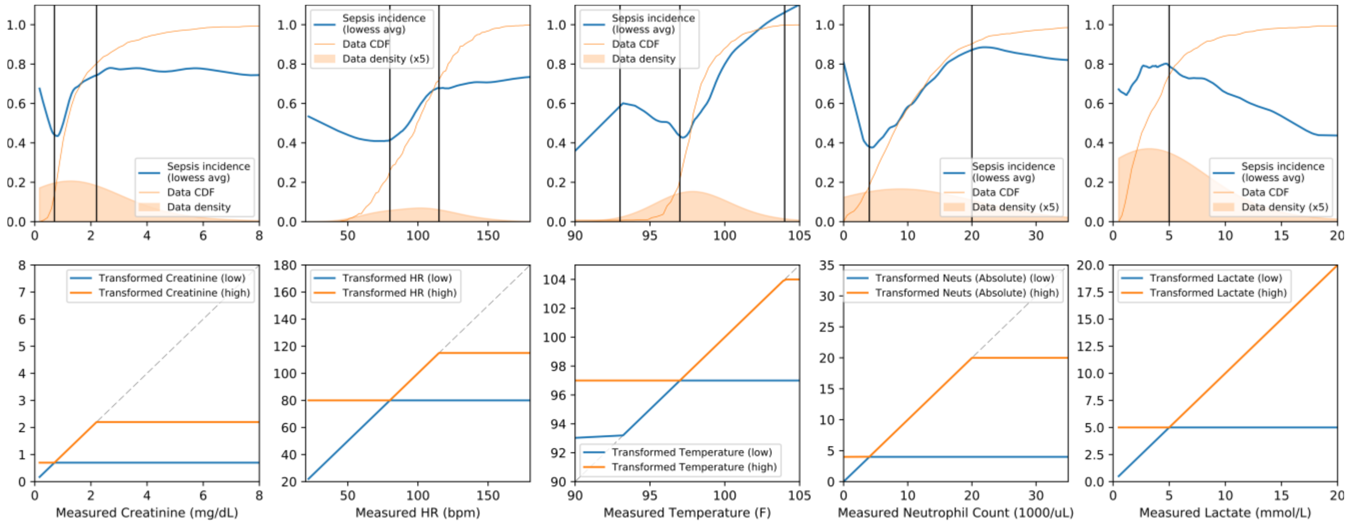

Fig SM-2: Transformations of continuous variables that were found to have biphasic associations with sepsis. Top row includes LOWESS regression with sepsis incidence, along with the cumulative distribution function of the variable at triage and a smoothed probability density function. Density functions that are very broad were multiplied by five for visualization purposed where noted. Vertical black lines indicate the thresholds below or above which variable values were clipped. Bottom row shows two lines, one for each of the two transformed variables created.

The full list of nonlinearly scaled continuous variables and their cut-offs are summarized in the table below.

Table SM-2. Nonlinearly scaled variables with cutoff values.

Variable		Cutoff(s)
SBP (mmHg)		100
SpO_2 (%)		90
Shock Index (bpm/mmHg)		0.65; 2.5
Pulse pressure (mmHg)		60
Respiratory rate (breaths/min)		16; 30
Heart rate (bpm)	high	80; 115
	low	80
Temperature (˚F)	high	97; 104
	low	93; 97

1.2. Adjudication of "auxiliary queries"

The **first** auxiliary query was "*Was there a report of a "bacterial infection symptom complex" (BISC)*?" *A priori*, our team developed criteria that we hypothesized would indicate that a patient likely has a bacterial infection based on symptoms alone. The intent of the BISC criteria was an objective clinical tool for determining whether a patient likely had a bacterial infection. The BISC was developed after prior work by our team on assessing clinical probability of infection, in which we noted excessive inter-rater variability. The BISC criteria are as follows:

- *BISC Criterion A:* At least one symptom *localizing* the source of infection;
- *BISC Criterion B*: At least one symptom indicative of an *infectious* process (constitutional or local inflammation);
- *BISC Criterion C: No alternative diagnosis* that was substantially more likely to cause those symptoms than bacterial infection.

In Table **SM-3**, we illustrate different subtypes of BISC that were also developed *a priori* to chart review.

Table SM-3. Bacterial infection symptom complex definitions.

**Symptom Complex**	**Localizing Symptom**	**Infectious Symptom (constitutional or local inflammation)**	**Exclusion Criteria** (i.e., *alternative diagnosis* that is substantially more likely to be causing symptoms than bacterial infection)
Bacterial Pneumonia	Productive cough, chest pain, dyspnea	Fever, fatigue/malaise, productive cough	Rhinorrhea, sinus congestion, aspiration, burn, trauma
Bacteria UTI	Flank pain, suprapubic pain, dysuria	Fever, fatigue / malaise, dysuria	Sudden onset of pain or hematuria suggestive of stone, trauma
Bacterial Abdominal	Abdominal pain / tenderness	Fever, fatigue / malaise, peritoneal signs	Trauma; or repeated emesis and diarrhea suggesting viral
Bacterial MSK	Pain / tenderness, erythema	Fever, fatigue / malaise, erythema, fluctuance / pus	Objective traumatic injury

After completing chart review for Interval-1, we examined the test characteristic of the BISC criteria for predicting sepsis. Recall that this was a study population of ED patients with at least one vital-sign abnormality documented during their ED visit (SBP < 100 mmHg or HR > SBP). For those patients who met criteria for any of the BISC subtypes, sensitivity for sepsis was 36% (28-45%); specificity was 95% (93-96%); and positive predictive value (PPV) was 62% (50-72%). Therefore, the majority of ED patients who had at least mild vital-sign abnormalities and who also met the BISC criteria were septic [1].

The **second** auxiliary query was "*Was there a report of fatigue or altered mental status*?" The **third** auxiliary query was "*Was there concern for bacterial infection prior to arrival in the Emergency Department?"* which included either referral from outpatient clinic with concern for infection, or report of fever/chills/rigors prior to arrival. Our guidelines for chart review for those questions are shown in Table **SM-4**.

Table SM-4. Criteria for chart review for "altered mental status", "fatigue/malaise", or "pre-arrival concern for infection".

"Altered mental status"	Was there written evidence to suggest the patient had altered mentation in the ED documentation (ED nursing notes, mid-level provider notes, MD notes)? In addition to the note(s) saying explicitly that the patient is confused or experiencing altered mental status, being somnolent, unresponsive and/or being found down (unless the patient has fallen due to weakness) also counts.
"Fatigue/Malaise"	If there is written evidence in the ED documentation of the patient being malaise/lightheaded/weak/lethargic you should check "yes" for this field.
"Pre-arrival concern for infection"	Referred in for infectious diagnosis or diagnostic data suggestive of infection? Not including explicitly viral processes such as diagnosed influenza. There must be labs that suggest infection or some other diagnostic data present, such as elevated WBC count, leukocytosis, positive blood culture, positive urine culture, chest x-ray findings (consolidation, PNA) or the patient was referred in for an explicit infectious diagnosis, such as sepsis, UTI, SBO, diverticulitis, etc.
"Fever/chills/rigors prior to ED arrival"	Was there written evidence to suggest the patient had fevers/chills/rigors prior to ED arrival? The fever can be a subjective fever and shaking can be considered chills if the patient doesn't have something else going on that could cause shaking. It's also important to note that "sweats" and "feels cold" doesn't count.

1.3. Adjudication of major comorbidities

Finally, we studied major comorbidities documented within the patient's medical record. "Any major comorbidity" for the Essential Model was taken as one or more from a list of major comorbidities. Criteria for the individual comorbidities is given in Table **SM-5**. In this analysis, these items were determined by manual chart review. In the future, the patient's EMR problem list could be electronically filtered to automatically determine whether such major comorbidities were present.

Table SM-5. Criteria for chart review for major comorbidities.

"active cancer"	If it's there has been resection/remission, and there is no current treatment (chemo, hormone-based chemo, radiation (XRT), trial drugs, ect.), then the cancer is not considered to be active. Active liquid tumors count for this field (e.g., myelodysplastic syndrome and multiple myeloma count).
"coronary artery disease"	Check "yes" for this field if there is written evidence in the admission note/discharge summary of past medical history of CAD, STEMI, NSTEMI, ischemic cardiomyopathy, or coronary atherosclerosis.
"congestive heart failure"	Check "yes for this field if there is written evidence in the admission note/discharge summary of past medical history of CHF, HF, HFpEF, HFrEF.
"COPD or chronic respiratory illness"	Check "yes" for this field if there is written evidence in the admission note/discharge summary of past medical history of COPD, home O2 use, interstitial lung disease, and chronic asthma.
"connective tissue disease"	You should mark this field as "yes" if there is evidence of SLE, polymyositis, mixed CTD, polymyalgia rheumatica, moderate to severe RA.
"cerebrovascular accident"	Check "yes" for this field if there is written evidence in the admission note/discharge summary of past medical history of CVA, ischemic CVA, hemorrhagic CVA, stroke.
"diabetes mellitus with end-stage complications"	None or uncomplicated for when the patient does not have diabetes, or if the patient does have diabetes but it is controlled by medication or diet. End-organ damage for when the patient has written evidence of end-organ dysfunction (e.g., retinopathy, neuropathy).
"immunocompromised"	If the patient is immunocompromised due to transplant, chronic immunosuppression medication, no spleen, AIDS, or primary immunodeficiency, check "yes" for this field.
"liver disease"	If there is no evidence of liver disease, select none. Check mild if there is cirrhosis without PHT, or if there are elevated LFTs with a history of HCV. Check moderate to severe if there is evidence of cirrhosis with portal hypertension +/- variceal bleeding.
"chronic disability"	Chronic disability due to quadriplegia, hemiplegia/paraplegia, dementia, inability to walk or care self, chronic trach/vent, tubes/drains, chronic wound, self-cath bladder.
"chronic kidney disease"	Check "yes" for this field if there is evidence of moderate CKD (creatinine >3mg/dL (0.27 mmol/L), or evidence of severe CKD (on dialysis, s/p kidney transplant, uremia).
"major surgery within one month"	Any incision or manipulation that went deeper than the skin and is more than a vascular-puncture/percutaneous. An example of just a vascular-puncture is a needle biopsy, stent, urinary stenting. Of note, I&D does count as it is more than a puncture and there is a possibility for pus re-accumulation.
"IV drug use"	Active IV drug use, not just history of IV drug use.

1.4. Characteristics of hospitals

Table SM-6. Emergency department characteristics of hospitals

**Hospital**	**Type**	**Emergency Department Volume (approximate)**	**Percentage of patients who received appropriate care for severe sepsis and/or septic shock, according to CMS SEP-1 measures***
Massachusetts General Hospital	Urban, academic	120,000	63%
Brigham and Women’s Hospital	Urban, academic	61,000 [2]	51%
North Shore Medical Center	Community	48,000 [3]	52%
Newton Wellesley Hospital	Community	55,000 [4]	55%

* CMS SEP-1 metrics data from: [5]; Massachusetts average for CMS SEP-1 metrics 55% and US National average 58%. Furthermore, not that the meaning of these measures remains controversial: “experts have continued to raise concerns that SEP-1 remains overly prescriptive, lacks a sound scientific basis and presents risks (overuse of antibiotics and inappropriate fluids not titrated to need).”. [6]

2. Supplementary results

2.1. Subject characteristics for additional hospitals

Table SM-7. Patient characteristics for additional validation cohorts. Values are presented as median (interquartile range) or proportion of cohort.

	NSMC		NWH		BWH	
	Non-sepsis Cases (N = 147)	Sepsis Cases (N =48)	Non-sepsis Cases (N = 141)	Sepsis Cases (N = 55)	Non-sepsis Cases (N = 122)	Sepsis Cases (N =74)
**Demographics**						
Age, years	63 (44, 79)	69 (61, 78)	59 (33, 82)	81 (69, 88)	58 (40, 70)	67 (56, 74)
Male, %	52	50	47	56	51	56
**Race**						
American Indian or Alaska Native, %	0	0	0	0	0	0
Asian, %	3	4	4	2	2	3
Black or African American, %	8	2	4	7	16	16
Middle Eastern or Northern African, %	0	0	0	0	0	0
Native Hawaiian or Pacific Islander, %	0	0	1	0	0	0
Other, %	3	0	5	2	11	7
White or Caucasian, %	84	94	87	89	66	73
Unavailable, %	1	0	0	0	4	1
**Ethnicity**						
Hispanic, %	12	15	5	2	14	8
Non-Hispanic, %	85	77	94	96	83	88
Unavailable, %	3	8	1	2	3	4
**Past medical history**						
Coronary artery disease, %	23.81	16.67	14.89	32.73	14.75	18.92
Congestive heart failure, %	19.73	27.08	17.73	25.45	16.39	17.57
Chronic kidney disease, %	18.37	33.33	12.77	29.09	12.30	20.27
Chronic obstructive pulmonary disease, %	19.73	29.17	7.09	14.55	13.11	21.62

Cerebrovascular accident, %	12.93	14.58	9.93	30.91	9.84	9.46
Liver disease, %	2.04	6.25	1.42	5.45	5.74	9.46

Table SM-8. Characteristics of Emergency Department presentation and outcomes for additional validation cohorts. Values are presented as median (interquartile range) or proportion of cohort.

	NSMC		NWH		BWH	
	Non-sepsis Cases (N = 147)	Sepsis Cases (N =48)	Non-sepsis Cases (N = 141)	Sepsis Cases (N = 55)	Non-sepsis Cases (N = 122)	Sepsis Cases (N =74)
**Vital signs**						
Triage SBP, mmHg	132 (114, 150)	103 (90, 132)	122.0 (103.0, 138.0)	101.0 (88.0, 138.5)	120 (105, 140)	102 (87, 122)
Proportion of patients w/ ED SBP <90 mmHg, %	24	58.3	26.95	67.27	24	70
Median time to hypotension from triage, min	81.0 (26.0, 209.5)	53 (10, 260)	77.5 (15.5, 161.25)	130.0 (26.0, 176.0)	34 (8, 55)	23.5 (9, 110.5)
Triage Heart rate, bpm	100.0 (86.5, 118.0)	102 (81, 130)	102.0 (86.0, 113.0)	100.0 (83.0, 121.0)	93 (76.25, 108)	108 (94.25, 123)
Triage GCS score	15 (15, 15)	15 (14, 15)	15 (15, 15)	15 (14, 15)	15 (15, 15)	15 (14, 15)
Triage respiratory rate, min^{-1}	18.0 (17.5, 22.0)	20 (18, 22)	20.0 (18.0, 24.0)	22.0 (20.0, 28.0)	18 (16, 20)	18 (18, 24)
Triage SpO_2, %	97.0 (94.0, 98.0)	95 (93, 96)	97.0 (96.0, 99.0)	95.0 (92.5, 97.0)	97.0 (95.0, 99.0)	96 (94, 98)
Triage temperature, ˚F	98.3 (97.6, 99.2)	100 (98, 102)	98.4 (98.0, 99.4)	99.1 (97.8, 101.8)	98.3 (97.8, 99.1)	98.7 (97.525, 100.5)
**ED diagnostics**						
First serum lactate, mmol/L	2.3 (2.0, 4.1)	2.95 (2.33, 3.57)	2.3 (1.35, 4.25)	2.8 (2.2, 4.2)	1.4 (1.1, 2.3)	2.8 (1.6, 4.1)
No Lactate, %	90	54	58.16	0	37	0
Sent < 1 hour, %	3	15	2.13	10.91	5	8
Sent 1 to 3 hrs, %	5	27	29.08	63.64	49	89
Sent ≥ 3 hours, %	2	4	10.64	25.45	9	3
White blood cell count, 1000/μL	11.3	15.37	10.26	15.21	11.92	10.71

	(8.94, 13.77)	(10.14, 20.89)	(7.65, 14.075)	(10.205, 22.715)	(6.22, 17.65)	(7.39, 14.78)
No WBC, %	9	0	12.77	0	0	4
Sent < 1 hour, %	42	33	19.15	18.18	18	18
Sent 1 to 3 hrs, %	41	65	56.03	69.09	57	60
Sent ≥ 3 hours, %	7	2	12.06	12.73	26	18
**Outcome**						
Infection Source, %						
Pulmonary	N/A	25.00	N/A	23.64	N/A	24.32
Urinary		25.00		34.55		27.03
Intraabdominal		12.50		12.73		14.86
Skin / soft tissue		10.42		10.91		8.11
Other		8.33		3.64		8.11
Unknown		20.83		18.18		24.32
Hospital mortality, %	2	12.5	4.96	18.18	3.28	18.92

2.2. Model composition (additional details)

Additional technical details about the model, including details about the pre-processing of the predictor variables, is available in the first author's doctoral thesis [7].

For the "Essential Model" the final input variables were, in order of weight, as follows: pre-ED concern for infection; major comorbidities; fatigue or confusion; bacterial symptom complex; respiratory rate; shock index; gender; age; temperature; GCS; SBP; SpO2. Using the testing dataset, the calibration of this model was evaluated:

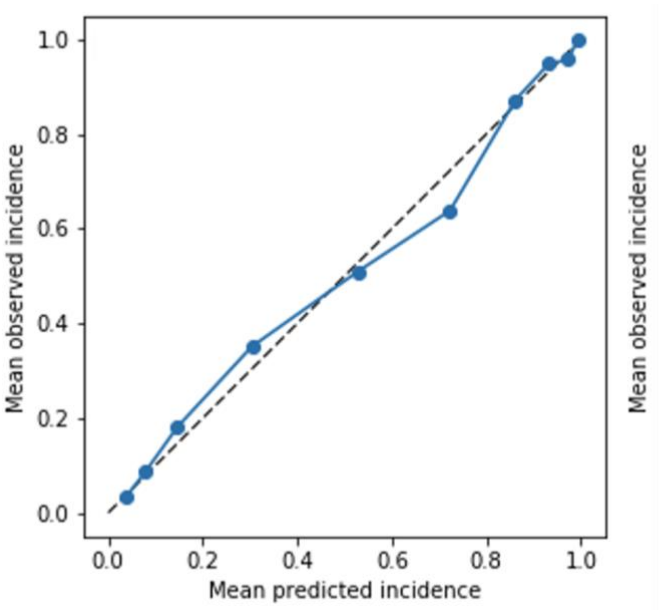

Fig SM-3: Calibration curve for Essential Model using testing dataset.

For the "Bland Model" the final input variables were, in order of weight, as follows: respiratory rate; shock index; temperature (fever only); age; gender; GCS; SBP; temperature (hypothermia only); and SpO2.

2.3. Audit for biases related to social determinants of health

In multivariable analysis of the relationship between the Essential Model output and sepsis, race/ethnicity and gender were not significant factors, i.e., $p > 0.05$, indicating that there was no statistically significant global bias towards positive predictions nor negative predictions by the Essential Model as a function of race/ethnicity nor gender. See Table SM-9.

Table SM-9. Multivariable analysis: Association between essential model output, race/ethnicity (i.e., White/non-Hispanic versus non-White or Hispanic); and gender (i.e., male versus non-male)

	P-value for essential model output (i.e., predicted probability of sepsis	P-value for race/ethnicity (White/non-Hispanic versus non-White or Hispanic)	P-value for gender (male versus non-male)
**MGH**	$P = 6.7\times10^{-9}$[a]	*P* = .19	*P* = .73
**BWH**	$P = 1.3\times10^{-11}$[b]	*P* = .96	*P* = .37
**NWH**	$P = 1.1\times10^{-6}$[c]	*P* = .76	*P* = .24
**NSMC**	$P = 2.9\times10^{-7}$[d]	*P* = .40	*P* = .75
**Pooled MGH, BWH, NWH, NSMC**	$P = 2.0\times10^{-31}$[e]	*P* = .26	*P* = .35

[a] Beta coefficient = 3.68; [b] Beta coefficient = 4.50; [c] Beta coefficient = 2.95; [d] Beta coefficient = 3.33; [e] Beta coefficient = 3.62.

There was also no association between Essential Model accuracy (i.e., incorrect prediction) as a function of race/ethnicity and gender, using the high-specificity cut-off (i.e., ≥ 0.6), except for one hospital “BWH” where there was a potentially significant association (*P* = 0.04) between accuracy and gender. Specifically, the prediction error rate was found to be *lower* for non-male patients and *higher* for male patient (logistic regression beta coefficient beta coefficient for male gender as a predictor of error = 3.62). See Table SM-10. We confirmed that male patients also had higher rates of false negatives and false positives in the BWH cohort.

Table SM-10. Multivariable analysis: Association between essential model error, race/ethnicity (i.e., White/non-Hispanic versus non-White or Hispanic); and gender (i.e., male versus non-male using high-specificity cutoff (i.e., ≥ 0.6)

	**P-value for race/ethnicity (White/non-Hispanic versus non-White or Hispanic)**	**P-value for gender (male versus non-male)**
**MGH**	*P* = .69	*P* = .80
**BWH**	*P* = .98	*P* = .04*
**NWH**	*P* = .40	*P* = .46
**NSMC**	*P* = .97	*P* = .90
**Pooled MGH, BWH, NWH, NSMC**	*P* = .56	*P* = .18

***** Higher rates of prediction error associated with male patients, driven by higher rates of both false positives and false negatives for the low-sensitivity cut-off.

There was potential association between Essential Model accuracy (i.e., incorrect prediction) as a function of race/ethnicity and gender, using the high-sensitivity cut-off (i.e., ≥ 0.2): the prediction error rate was found to be *lower* for non-white or Hispanic patients and higher for white/non-Hispanic patients. Also, the prediction error rate was found to be *lower* for non-male patients and higher for male patients. See Table SM-11. We examined the data underlying these findings and confirmed that white patients had higher rates of false negatives and also false positives when using the high-sensitivity cut-off (i.e., ≥ 0.2). We further confirmed that male patients also had higher rates of false negatives and false positives. This analysis was done as an audit to ensure that there was no important bias associated with social determinants of health, and to this end, it is reassuring that we did not find prediction errors associated with under-represented minorities or non-male patients. The specific interpretation is uncertain and may be a function of multiple comparisons, because after Bon Ferroni correction for the multiple comparisons done for this audit, the P-values are no longer statistically significant.

Table SM-11. Multivariable analysis: Association between essential model error, race/ethnicity (i.e., White/non-Hispanic versus non-White or Hispanic); and gender (i.e., male versus non-male) using high-sensitivity cutoff (i.e., ≥ 0.2)

	**P-value for race/ethnicity (White/non-Hispanic versus non-White or Hispanic)**	**P-value for gender (male versus non-male)**
**MGH**	*P* = .42	*P* = .65
**BWH**	*P* = .25	*P* = .14
**NWH**	*P* = .40	*P* = .46
**NSMC**	*P* = .21	*P* = .14
**Pooled MGH, BWH, NWH, NSMC**	*P* = 0.03*	*P* = 0.04*

***** Higher rates of prediction error associated with white patients and also with male patients, driven by higher rates of both false positives and false negatives for the high-sensitivity cut-off.

3. References

1. Biebelberg B, Prasad V, Lynch JC, Nepal S, Filbin MR, Heldt T, et al. Bacterial infection symptom complex criteria: predicting sepsis from the history of present illness. The society of academic emergency medicine annual meeting; 2020 May; Virtual Location (COVID-19)2020. p. s195.
2. MassGeneralBrigham. Brigham and women's hospital department of emergency medicine [Internet] [cited 07/03/2023]. Available from: https://www.brighamandwomens.org/emergency-medicine#:~:text=We%20care%20for%20nearly%2060%2C000,of%20Massachusetts%2C%20and%20New%20England.
3. Patient CareLink. Salem hospital - emergency department [Internet] 2013 [cited 07/03/2023]. Available from: http://data.patientcarelink.org/staffing2013/edrick.cfm?hid=69&ID=8989&Name=Salem%20Hospital&Unit=Emergency%20Department.
4. MassGeneralBrigham. Newton wellesley hospital facts & figures [Internet] 2021 [cited 07/03/2023]. Available from: https://www.nwh.org/about-us/facts-figures.
5. Medicare. Massachusetts general hospital [Internet]: Medicare.gov; [updated April 26, 2023; cited June 28, 2023]. Available from: https://www.medicare.gov/care-compare/details/hospital/220071?city=Boston&state=MA&zipcode=02114.
6. Wang J, Strich JR, Applefeld WN, Sun J, Cui X, Natanson C, et al. Driving blind: instituting SEP-1 without high quality outcomes data. J Thorac Dis. 2020;12(Suppl 1):S22-S36. doi: 10.21037/jtd.2019.12.100. PubMed PMID: 32148923; PubMed Central PMCID: PMCPMC7024755.
7. Prasad V. Learning from clinical health data for real-time decision support in emergency department care of sepsis. PhD. Thesis, Massachusetts Institute of Technology; 2019.
